# Acute erythroblastopenia due to Parvovirus B19 in hemoglobinopathies: a retrospective case series at Ibn Sina Hospital, Rabat, Morocco

**DOI:** 10.11604/pamj.2026.54.2.49819

**Published:** 2026-05-06

**Authors:** Ismail Regragui, Hassane Mamad, Jalila Zirar, Najoua El Mokhtari, Mohamed Ifleh, Souad Benkirane, Azlarab Masrar

**Affiliations:** 1 Central Hematology Laboratory, Ibn Sina Hospital Center, Rabat, Morocco,; 2 Hematology Laboratory, Faculty of Medicine and Pharmacy, Mohammed V University, Rabat, Morocco

**Keywords:** Parvovirus B19, acute erythroblastopenia, hemoglobinopathies, sickle cell disease, children, Morocco

## Abstract

This study describes the clinical and hematological characteristics of acute erythroblastopenia induced by Parvovirus B19 in patients with underlying hemoglobinopathies. We conducted a retrospective analysis of 53 patients with confirmed Parvovirus B19 infection, among whom 10 pediatric cases met the criteria for acute erythroblastopenia, defined by hemoglobin <6 g/dL and reticulocytopenia <20x10^9^/L. Among 53 patients with confirmed Parvovirus B19 infection, 10 pediatric cases (18.9%) met the criteria for acute erythroblastopenia. All cases occurred in children with various hemoglobinopathies: sickle cell disease (3 cases), S/beta-thalassemia (1 case), hemoglobin C disease (2 cases), and hereditary spherocytosis (2 cases), while two patients had no identified hemoglobinopathy. The mean hemoglobin was 5.13 ± 1.66 g/dL, with profound reticulocytopenia. Multi-lineage involvement occurred in 20% (n=2/10) of cases. Bone marrow examination showed variable erythroblast percentages (1-47%). Recovery time ranged from 6 to 35 days, with the longest recovery observed in sickle cell patients. Parvovirus B19 causes severe erythroblastopenic crises in children with hemoglobinopathies, particularly in sickle cell disease, where the clinical impact is most pronounced.

## Introduction

Acute erythroblastopenia is a serious hematological complication characterized by the sudden arrest of bone marrow erythroid precursor production. This phenomenon results from selective damage to the red blood cell lineage, leading to a temporary but profound inability of the bone marrow to generate new erythrocytes. Among its principal causes, Parvovirus B19 infection holds a particular place due to its specific tropism for erythroblasts [1]. This single-stranded DNA virus, extremely widespread in the general population, most often causes asymptomatic or benign infections manifesting as erythema infectiosum (fifth disease) in children or arthralgias in adults. Its transmission, primarily respiratory, explains its high transmissibility and worldwide distribution. However, the clinical presentation takes on a completely different dimension in patients with heightened erythropoiesis, as seen in inherited or acquired hemolytic hemoglobinopathies. In these patients, who already have increased red blood cell turnover, the infection triggers particularly severe aplastic crises. The combination of chronic hemolysis and complete bone marrow arrest leads to a sharp drop in hemoglobin (often <4 g/dL) with profound reticulocytopenia. These transient episodes constitute genuine hematological emergencies, requiring immediate and specialized management [2]. Recognizing this specific clinical picture and understanding its underlying mechanisms are essential for any clinician managing patients with hemoglobinopathies. Early diagnostic suspicion indeed allows for the prompt initiation of appropriate therapeutic measures, including red blood cell transfusion and, in some cases, administration of intravenous immunoglobulins [3]. This study aimed to evaluate the impact of Parvovirus B19 on the severity of erythroblastopenic crises in patients with chronic hemolytic anemias, with particular attention to differences in severity according to the underlying pathologies.

## Methods

**Study design and setting:** this retrospective study was conducted at the Central Hematology Laboratory of Ibn Sina Hospital Center, Rabat, Morocco, between August 2022 and November 2024.

**Study population:** the target population included all patients with confirmed Parvovirus B19 infection during the study period. Inclusion criteria were: age < 18 years, confirmed Parvovirus B19 infection by positive IgM serology and/or PCR, and acute erythroblastopenia defined as hemoglobin < 6 g/dL and reticulocyte count < 20×10^9^/L. Patients with incomplete medical records were excluded. Among 53 patients with confirmed Parvovirus B19 infection, 10 pediatric cases met the inclusion criteria and were included in the analysis. No sample size estimation was performed due to the descriptive nature of this case series.

**Data collection:** data were collected retrospectively from medical records during routine follow-up visits. For each patient, we recorded hematological parameters including hemoglobin level, reticulocyte count, and erythroblast percentage on bone marrow examination. Associated cytopenias (thrombocytopenia, leukopenia) were also documented. Underlying hemoglobinopathies were diagnosed by High-Performance Liquid Chromatography (HPLC) for sickle cell disease, thalassemias, and hemoglobin C disease, supplemented by an osmotic fragility test for hereditary spherocytosis. Parvovirus B19 infection was confirmed by serology (IgM) and/or PCR.

**Definitions:** acute erythroblastopenia was defined as hemoglobin < 6 g/dL associated with reticulocytopenia < 20x10^9^/L. Multi-lineage involvement was defined as the presence of thrombocytopenia (platelet count < 150x10^9^/L) and/or leukopenia (white blood cell count < 4.0x10^9^/L) in addition to anemia. Time to hematological recovery was defined as the interval between diagnosis and the return of hemoglobin to ≥8 g/dL with a reticulocyte count >50x10^9^/L.

**Statistical analysis:** data were analyzed using descriptive statistics. Continuous variables were expressed as mean ± standard deviation. Categorical variables were presented as frequencies and percentages. Due to the small sample size, no comparative statistical tests were performed. All analyses were conducted using Microsoft Excel.

**Ethical considerations:** the study protocol was approved by the Ethics Research Committee of Ibn Sina Hospital Center (approval number: CERB-IS-2023-045). The study was conducted in accordance with ethical standards. Patient data were anonymized, and confidentiality was maintained throughout the study. Informed consent was waived due to the retrospective nature of the study.

## Results

Among the 53 patients infected with Parvovirus B19, 10 pediatric cases (18.9%) met the criteria for acute erythroblastopenia, defined by hemoglobin <6 g/dL and reticulocytes <20×10^9^/L. In these 10 patients, HPLC investigations identified three cases of homozygous sickle cell disease, one double heterozygosity for S/beta-thalassemia, and two cases of hemoglobin C disease, while two cases of hereditary spherocytosis were diagnosed by osmotic fragility test, and two other cases had no underlying hemoglobinopathy. The direct antiglobulin test (Coombs test) was negative for all ten patients, ruling out an associated immune component. In this series, severe anemia was manifested by a mean hemoglobin level of 5.13 ± 1.66 g/dL and marked reticulocytopenia at 16.3 ± 24.22 G/L, indicating severe erythroid medullary insufficiency. Hematological evaluation revealed two distinct profiles: seven cases of normochromic normocytic anemia and three cases of hypochromic microcytic anemia. Bone marrow aspirate revealed variable residual erythropoiesis: four patients presented 1%, 1%, 4%, and 7% erythroblasts, respectively, while the other six showed rates between 12% and 47%. All hematological data are summarized in Table 1.

**Table 1:** hematological characteristics of patients according to the type of hemoglobinopathy

Hemoglobinopathy	Number of patients	Mean Hb (g/dL)	Mean reticulocytes (G/L)	Bone marrow erythroblasts (%)
Sickle cell disease	3	3.4	5.3	1%, 1%, 4%
S/beta-thalassemia	1	4.9	17.7	7%
Hereditary spherocytosis	2	5.5	18.8	12%, 19%
Hemoglobin C disease	2	5.7	19.5	23%, 38%
No identified hemoglobinopathy	2	6	20	31%, 47%
Overall mean/total	10	5.13	16.3	

Hb: hemoglobin

The study highlights a correlation between the type of hemoglobinopathy and the severity of the clinical presentation. The lowest hemoglobin values were observed in sickle cell patients, with a nadir of 2.8 g/dL and marked reticulocytopenia reaching 2 G/L. The other hemoglobinopathies showed a decreasing gradation of severity: S/beta-thalassemia, followed by hereditary spherocytosis, and finally hemoglobin C disease. In this series of 10 patients with erythroblastopenia, associated thrombocytopenia was documented in 3 cases, with values ranging from 17 to 60 G/L. Two of these patients presented with bicytopenia, combining thrombocytopenia and leukopenia (2.1 and 1.8 G/L, respectively), while the third maintained a normal white blood cell count.

Two intrafamilial cases of erythroblastopenia were identified, involving a boy (Hb 4.9 g/dL; reticulocytes 5 G/L) and his sister (Hb 3.7 g/dL; reticulocytes 4 G/L). Although the female patient presented with more profound anemia (difference of 1.2 g/dL), her kinetics of hematological normalization proved faster than that of her brother. Recovery from the erythroblastopenic period required a duration between 6 and 35 days in our 10 patients. Sickle cell patients presented the longest recovery delays, with the maximum duration observed (35 days) in one of them, while the other pathologies showed faster recoveries.

## Discussion

This study aimed to evaluate the impact of Parvovirus B19 on the severity of erythroblastopenic crises in patients with chronic hemolytic anemias, with particular attention to differences according to the underlying hemoglobinopathy. The main findings show that all 10 cases of acute erythroblastopenia occurred exclusively in children, with a clear severity gradient ranging from sickle cell disease (lowest hemoglobin: 2.8 g/dL, reticulocytes: 2 G/L, longest recovery: 35 days) to hemoglobin C disease (milder presentation). Multi-lineage involvement was observed in 20% of cases, and bone marrow examination revealed highly variable residual erythropoiesis ranging from 1% to 47% erythroblasts.

Parvovirus B19 is a ubiquitous human pathogen whose seroprevalence increases with age, reaching 15-60% in adolescents and up to 80% in adults [1]. In our study, the observed cases of erythroblastopenia concerned exclusively children managed in pediatric services. Transmitted mainly via the airborne route, and more rarely through blood exposure, the virus reaches the bone marrow where it specifically targets erythroid precursors via the globoside receptor (P antigen) [4,5]. The clinical expression of Parvovirus B19 infection varies significantly depending on the patient's hematological status. In subjects with hemoglobinopathies and chronic hemolysis, the virus causes a sudden and complete inhibition of erythropoiesis, provoking acute erythroblastopenia of particular severity. This clinical picture results from a dual pathogenic mechanism: on the one hand, the persistence of underlying hemolysis, on the other hand, the complete arrest of medullary erythroid production [6]. This synergy explains the extreme hemoglobin drops (reaching 2.8 g/dL in our cohort) and profound reticulocytopenia (<10 G/L) that constitute the characteristic biological markers of these aplastic crises.

**Figure 1:**
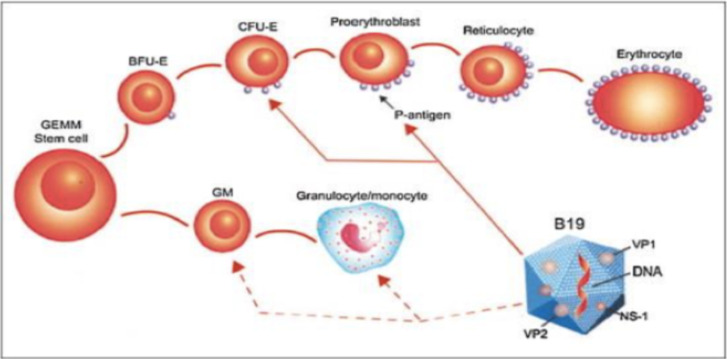
cellular targets of Parvovirus B19: binding to globoside and effects on erythroid (CFU-E), myeloid (CFU-GM), and megakaryocytic (CFU-Meg) progenitors

The non-structural protein NS1 of Parvovirus B19 exerts multisystemic cytotoxic effects demonstrated in vitro. Beyond its well-characterized inhibitory action on erythroid precursors (cell cycle blockade and apoptosis induction), fundamental studies, notably by Hanada *et al*. have revealed its impact on myeloid (CFU-GM) and megakaryocytic (CFU-Meg) lineages. In our study, this multi-lineage involvement was observed in two patients presenting simultaneously with leukopenia and thrombocytopenia, confirming the broader cytotoxic effect of the NS1 protein on multiple hematopoietic lineages, as illustrated in Figure 1 [7-9].

The causal relationship between Parvovirus B19 infection and erythroblastopenic crises was first established in 1981 in sickle cell patients. This initially described pathogenic association was subsequently strongly supported by work in Jamaica, where retrospective analysis of sera demonstrated that 86% of these hematological crises resulted from recent infection with this virus [10]. The hematological parameters of our sickle cell patients reveal a minimum hemoglobin of 2.8 g/dL, slightly lower than the 3.1 g/dL reported by Serjeant *et al*. associated with measurable reticulocyte counts (2-7 G/L), contrasting with the complete reticulocytopenia (<0.1%) typically observed [2].

Our study highlights significant variability in bone marrow suppression during Parvovirus B19-induced erythroblastopenia. Among the 10 analyzed cases, we observe two distinct profiles: four patients had extremely low erythroblast counts (1%, 1%, 4%, and 7%), indicating near-complete inhibition of erythropoiesis, while six patients retained partial medullary activity (rates between 12% and 47%). This heterogeneity could be explained by several factors. On one hand, variability in the timing of sampling relative to the onset of infection can explain part of this medullary heterogeneity, as the stage of infection at which bone marrow is examined significantly influences the observed cytological picture [11]. This disparity is also explained by the particular hematological context of these patients, whose underlying chronic hemolysis maintains the bone marrow in a state of permanently accelerated erythropoiesis. On the other hand, individual differences in immune response or viral load could also modulate the severity of the medullary involvement [12].

Patients with increased erythropoiesis, whether of constitutional or acquired origin, are particularly vulnerable to the hematological complications of Parvovirus B19. The main pathologies concerned include hemolytic hemoglobinopathies (sickle cell disease, thalassemias, hemoglobin C disease), red blood cell membrane abnormalities (hereditary spherocytosis, elliptocytosis), and enzymatic deficiencies (pyruvate kinase). Acquired forms, such as autoimmune hemolytic anemias and paroxysmal nocturnal hemoglobinuria, are also at risk. It should also be noted that this complication is not limited to constitutional or acquired hemolytic diseases alone. It can indeed occur in various states of erythropoietic stress, notably during acute or chronic hemorrhage, in cases of severe iron deficiency anemia, or in the post-transplantation (renal or medullary) setting, situations where the bone marrow is particularly solicited [13]. This may explain the two parvovirus B19-positive cases without an identified hemoglobinopathy, who likely experienced transient erythroblastopenia due to an unrecognized bone marrow stressor, as previously described in immunocompetent children [14]. Although rare, simultaneous familial cases of erythroblastopenia have been occasionally described in the literature. We report here the particular observation of a sibling of two patients presenting with acute erythroblastopenia related to Parvovirus B19. This case perfectly illustrates the particularly efficient interhuman transmission of this virus, mainly airborne, explaining the simultaneous occurrence of the infection in these two relatives [15].

**Figure 2:**
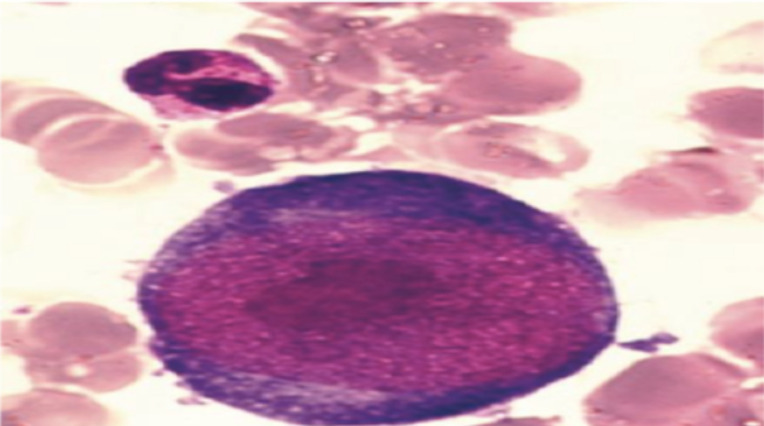
giant proerythroblasts observed during the acute phase of Parvovirus B19 infection

During the acute phase of Parvovirus B19 infection, bone marrow examination classically reveals giant proerythroblasts, pathognomonic elements resulting from the direct cytopathic effect of the virus. However, this typical cytological characteristic could not be observed in the bone marrow aspirates performed in our patients, despite biological confirmation of the infection (Figure 2) [16]. Certain immunodeficiencies, particularly those affecting cellular immunity, predispose to chronic medullary infections with Parvovirus B19. These infections are characterized by severe regenerative anemia resulting from persistent erythroblastopenia. In these specific cases, detection of the viral genome by PCR in bone marrow represents the diagnostic method of choice, due to the patient's inability to mount an adequate antibody response. The first case described in the literature dates back to 1984 and concerned a child with severe combined immunodeficiency (Nezelof syndrome). This initial description definitively established the link between cellular immunodeficiency and persistence of medullary viral infection. In these particular clinical situations, the chronic course of the infection generally requires treatment with specific intravenous immunoglobulins (IVIg) to compensate for the immune deficit [17].

The normal recovery of erythropoiesis after Parvovirus B19 infection occurs through the elimination of the virus by the humoral immune response, with the progressive reappearance of normal erythroid precursors in the bone marrow and the disappearance of the giant proerythroblasts characteristic of acute infection. This medullary reconstitution is accompanied by an increase in blood reticulocytes within 1 to 2 weeks, leading to complete normalization of hematologic parameters [2].

These findings have several clinical implications. In any child with a known hemoglobinopathy presenting with acute anemia and reticulocytopenia, Parvovirus B19 infection should be the primary diagnostic hypothesis [10]. As demonstrated by Serjeant *et al*. and Pattison *et al*. this virus is responsible for the majority of aplastic crises in patients with sickle cell disease, and delayed recognition may lead to life-threatening complications [2,10]. The severity gradient observed across different hemoglobinopathies can guide clinicians in anticipating the need for transfusion support: patients with sickle cell disease are at the highest risk and require the most urgent intervention, as illustrated by the prolonged recovery time in this subgroup. The absence of giant proerythroblasts on bone marrow examination should not rule out the diagnosis, particularly if sampling occurs later in the course of infection [11]. In such cases, PCR confirmation remains essential for diagnosis [11]. Furthermore, the occurrence of familial cases underscores the importance of screening siblings when a case is diagnosed, especially in households with multiple children with hemolytic conditions [14].

This study has several limitations, including its retrospective design, the absence of viral quantification, the heterogeneity in the timing of bone marrow examinations, and the lack of precise evaluation of therapeutic interventions. The small sample size did not allow for statistical comparisons between subgroups. Although giant proerythroblasts typically represent a characteristic marker of this infection, they were not observed in our series. However, the study also has notable strengths: a homogeneous definition of erythroblastopenia, the use of standardized diagnostic methods (HPLC, osmotic fragility, Coombs test), systematic bone marrow examination in all patients, and detailed characterization of hematological parameters across different hemoglobinopathy types, providing a valuable reference for clinicians in similar settings.

## Conclusion

This study shows that Parvovirus B19 is a cause of severe erythroblastopenia in children with hemoglobinopathies. A severity gradient was observed, ranging from sickle cell disease (lowest hemoglobin: 2.8 g/dL, longest recovery: 35 days) to hemoglobin C disease (milder forms). Multi-lineage involvement occurred in two patients, and bone marrow examination revealed variable erythroblast counts. These findings highlight the importance of considering Parvovirus B19 infection in any child with hemoglobinopathy presenting with acute anemia and reticulocytopenia.

### What is known about this topic

- Parvovirus B19 is a recognized cause of acute erythroblastopenia in immunocompetent individuals;
- Patients with chronic hemolytic anemias are particularly vulnerable to severe aplastic crises from B19 infection;
- The association between Parvovirus B19 and sickle cell disease complications is well-established.

### What this study adds

- First analysis of Parvovirus B19-induced erythroblastopenia across hemoglobinopathies in Moroccan children;
- A clear severity gradient was established, ranging from sickle cell disease to hemoglobin C disease;
- Multi-lineage involvement confirms the broader cytotoxic effect of the virus beyond the erythroid lineage.
